# Pain-related stress during the Neonatal Intensive Care Unit stay and *SLC6A4* methylation in very preterm infants

**DOI:** 10.3389/fnbeh.2015.00099

**Published:** 2015-04-21

**Authors:** Livio Provenzi, Monica Fumagalli, Ida Sirgiovanni, Roberto Giorda, Uberto Pozzoli, Francesco Morandi, Silvana Beri, Giorgia Menozzi, Fabio Mosca, Renato Borgatti, Rosario Montirosso

**Affiliations:** ^1^0-3 Center for the Study of Social Emotional Development of the at-Risk Infant, Scientific Institute, IRCCS Eugenio MedeaBosisio Parini (LC), Italy; ^2^NICU, Department of Clinical Sciences and Community Health, Università degli Studi di Milano, Fondazione IRCCS Ca' Granda Ospedale Maggiore PoliclinicoMilan, Italy; ^3^Molecular Biology Lab, Scientific Institute, IRCCS Eugenio MedeaBosisio Parini (LC), Italy; ^4^Bioinformatic Lab, Scientific Institute, IRCCS Eugenio MedeaBosisio Parini (LC), Italy; ^5^Pediatric Unit, Sacra Famiglia HospitalErba (CO), Italy; ^6^Neuropsychiatry and Neurorehabilitation Unit, Scientific Institute, IRCCS Eugenio MedeaBosisio Parini (LC), Italy

**Keywords:** DNA methylation, Neonatal Intensive Care Unit, pain-related stress, preterm infants, serotonin, *SLC6A4* gene

## Abstract

Very preterm (VPT) infants need long-lasting hospitalization in the Neonatal Intensive Care Unit (NICU) during which they are daily exposed to pain-related stress. Alterations of DNA methylation at the promoter region of the *SLC6A4* have been associated with early adverse experiences in infants. The main aim of the present work was to investigate the association between level of exposure to pain-related stress during hospitalization and changes in *SLC6A4* DNA methylation at NICU discharge in VPT infants. In order to exclude the potential effect of birth status (i.e., preterm vs. full-term birth) on *SLC6A4* methylation, we preliminarily assessed *SLC6A4* epigenetic differences between VPT and full-term (FT) infants at birth. Fifty-six VPT and thirty-two FT infants participated in the study. The level of exposure to pain-related stress was quantified on the basis of the amount of skin-breaking procedures to which they were exposed. VPT infants were divided in two sub-groups: *low-pain exposure* (LPE, *N* = 25) and *high-pain exposure* (HPE, *N* = 31). DNA methylation was evaluated at birth for both VPT and FT infants, assessing 20 CpG sites within the *SLC6A4* promoter region. The same CpG sites were re-evaluated for variations in DNA methylation at NICU discharge in LPE and HPE VPT infants. No differences in *SLC6A4* CpG sites' methylation emerged between FT and VPT infants at birth. Methylation at CpG sites 5 and 6 significantly increased from birth to NICU discharge only for HPE VPT infants. Findings show that preterm birth *per se* is not associated with epigenetic alterations of the *SLC6A4*, whereas higher levels of pain-related stress exposure during NICU stay might alter the transcriptional functionality of the serotonin transporter gene.

## Introduction

Preterm birth represents a major health care issue worldwide with the rate of preterm birth ranging from about 5% in some European countries to 18% in several African countries (Blencowe et al., 2013). During last decades, medical progresses have contributed to grant higher survival rates even for infants born at very low gestational age (i.e., 24–32 weeks gestational age) (Hack and Fanaroff, 1999). Nonetheless, even in absence of major post-natal morbidities and brain injuries, very preterm (VPT) infants are neuro-behaviorally immature and require long-lasting hospitalization in the Neonatal Intensive Care Unit (NICU) (Lester et al., 2011a; Grunau, 2013).

It has been shown that VPT infants are exposed throughout the course of hospitalization in the NICU to stressful experiences which are mainly represented by painful interventions (e.g., skin-breaking procedures) (Vinall et al., 2012; Vinall and Grunau, 2014). As stress and pain cannot be easily distinguished in immature infants (Chau et al., 2014), it seems appropriate to acknowledge that VPT infants experience pain-related stress during the neonatal period, which is characterized by a rapid brain development and programming of stress systems (Lester et al., 2011a; Hunter, 2012; Ranger et al., 2013). Several studies have documented that reiterated exposure to high levels of pain-related stress can induce long-lasting potential effects on brain organization and neuroendocrine response to stress (Grunau et al., 2005; Smith et al., 2011; Grunau, 2013). Repeated pain-related stress might lead to phenomenon of sensitization in VPT infants, resulting in an ongoing dysregulation of stress response systems at short- (i.e., during the NICU stay) and long-term (i.e., infancy and childhood). Higher levels of pain-related stress (e.g., numbers of neonatal skin-breaking procedures) have been associated with reduced development of white matter and subcortical gray matter (Brummelte et al., 2012), motor and cognitive development in preschoolers (Grunau et al., 2009), altered activity of hypothalamic-pituitary-adrenal (HPA) axis (Grunau et al., 2013) and internalizing behavioral symptoms during school age (Ranger et al., 2013) in VPT infants, after adjusting for specific medical confounders.

The serotoninergic system is one of the major brain systems associated with stress regulation and is affected by exposures to early adversity (Lesch, 2011). The serotonin fibers are widely distributed throughout the central nervous system, they appear early during gestation and rapidly develop during infancy (Canli and Lesch, 2007). The serotoninergic system is regulated by feedback mechanisms through the serotonin transporter (5-HTT), which is a protein encoded by the *SLC6A4* gene. This gene has structural genetic variants (i.e., the 5-HTTLPR polymorphism): a short and a long allelic variant. The short variant results in a two-fold reduction of serotonin reuptake compared to the long one (Canli and Lesch, 2007) and has been associated with heightened stress vulnerability and poorer socio-emotional outcomes during infancy and childhood (Pauli-Pott et al., 2009). Moreover, the transcriptional activity of the *SLC6A4* gene has been found to be regulated by epigenetic mechanisms (Lesch, 2011; Mueller et al., 2011; Griffiths and Hunter, 2014). The methylation status of different CpG sites within the *SLC6A4* promoter region has been inversely related to the degree of serotonin transporter expression (Philibert et al., 2007, 2008). Early adverse experiences, such as abuse during childhood in adopted children (Beach et al., 2010, 2011) and the exposure to maternal depression during pregnancy (Devlin et al., 2010) have been found to associate with patterns of altered methylation at the level of different CpG sites within the *SLC6A4* promoter region. Hence, the *SLC6A4* gene is suggested to be a key epigenetic regulator of the impact of early adverse experiences on subsequent development of human infants and children.

Although the epigenetic study of early adverse experiences in preterm birth and pain-related stress is strongly suggested (Lester et al., 2011b; Samra et al., 2012; Maddalena, 2013), there is a paucity of epigenetic studies investigating the methylation status of stress-related genes in young infants born prematurely (Montirosso and Provenzi, 2015). Recently, it has been shown that early and repeated exposure to skin-breaking procedures might be predictive of altered methylation at the level of *SLC6A4* (Chau et al., 2014). A retrospective study has investigated the role of *SLC6A4* methylation in relation to both pain-related stress during NICU stay and behavioral problems in 7-year-old children (Chau et al., 2014). The findings documented that higher exposure to pain-related stress during hospitalization was associated with higher methylation at 7/10 CpG sites in the *SLC6A4* promoter compared to full-term controls. Moreover, higher *SLC6A4* DNA methylation was found to be predictive of higher behavioral problems in VPT infants. In another study, no differences were found in the methylation of *SLC6A4* promoter region between full-term and preterm infants at birth (Montirosso et al., 2015a). Nonetheless, methylation increased from birth to NICU discharge at two specific CpG sites for preterm infants and the increased methylation was predictive of temperamental difficulties for these infants at 3-month-age.

Overall, these findings suggest that the exposure to repeated pain-related stress during the perinatal period is an early adverse experience that might associate with altered methylation of the *SLC6A4* promoter in VPT infants at short- and long-term. Unfortunately, to the best of our knowledge, no prospective studies have yet investigated the association between level of pain-related stress and *SLC6A4* methylation during the neonatal period, adopting a micro-longitudinal design. Examining the association between pain-related stress and epigenetic differences at specific CpG sites of the *SLC6A4* promoter in VPT infants might add to our understanding of how early adverse experiences can affect the neurodevelopment of preterm infants, via functional modifications of the serotoninergic system.

In the current study, we investigated the relationship between the level of pain-related stress and *SLC6A4* epigenetic changes at NICU discharge in VPT infants. We categorized VPT infants into two groups (i.e., *low-pain exposure* and *high-pain exposure*) on the basis of the amount of skin-breaking procedures to which they were exposed during NICU stay. We examined if different levels of pain-related stress were associated with changes of *SLC6A4* methylation status (controlled for clinical confounders) from birth to NICU discharge. In order to control for the potential effect of birth status (i.e., preterm *vs*. full-term birth), we first compared epigenetic differences in the *SLC6A4* methylation status between *low-pain exposure*, *high-pain exposure* and a group of full-term infants at birth. As no previous research directly assessed the association between levels of pain-related stress and *SLC6A4* methylation status at NICU discharge in VPT infants, no specific hypotheses were formulated. Nonetheless, consistently with previous literature on epigenetic correlates of early adverse experiences in infancy (Devlin et al., 2010), we speculated that infants exposed to higher pain-related stress would exhibit greater changes in *SLC6A4* methylation. Furthermore, given that *SLC6A4* methylation variations were expected to be associated with pain-related stress, we hypothesized no difference in methylation status at birth between VPT and full-term infants.

## Materials and methods

### Participants

A cohort of 88 infants were consecutively recruited between October 2011 and April 2014. Fifty-six VPT infants (27 females) were enrolled at the NICU of the Department of Clinical Sciences and Community Health, Fondazione IRCCS Ca' Granda Ospedale Maggiore Policlinico of Milan. Inclusion criteria for preterm infants were: gestational age < 32 weeks and/or birth weight ≤ 1500 g. Exclusion criteria included: major brain lesions as documented by cerebral ultra-sound (intraventricular hemorrhage > grade 2 according to Papile, cystic periventricular); neuro-sensorial deficits (rethinopathy of prematurity ≥ stage 2); genetic syndromes and/or major malformations. A group of 32 full-term infants (gestational age ≥ 37 weeks and birth weight ≥ 2500 g, 15 females) was recruited at the Pediatric Unit of the Sacra Famiglia Hospital, Erba (CO). Full-term (FT) infants were healthy, with no neonatal morbidities or prenatal/perinatal-risk factors. To obtain a homogenous group of mothers for both groups, we applied the following inclusion criteria: age over 18 years, good comprehension of Italian language, no obvious cognitive impairments, no manifest psychiatric disorders, no psychotropic medication during pregnancy, no prenatal depression or anxiety symptomatology, no drug addiction and no single-parent families. All parents of infants enrolled in the study signed a written informed consent form. The study was approved by the Ethics Committees of the Scientific Institute, IRCCS Eugenio Medea, Bosisio Parini (LC) and of the participating hospitals.

### Procedure

Adopting procedures from previous research in preterm behavioral epigenetics (Kantake et al., 2014), cord blood was collected at birth for both VPT and FT groups and, only for VPT infants, a peripheral blood sample was collected at hospital discharge, following routine clinical procedures. Blood samples were obtained by trained nurses to avoid hemolysis and immediately stored at −20°C.

### Measures

#### Socio-demographic and neonatal data

Socio-demographic data such as maternal age and occupational status of both parents were obtained. According to Hollingshead's classification (Hollingshead, 1975), the more prestigious occupational level between parents was considered as family socio-economic status (SES) score ranging from 0 to 90. Lower scores reflected lower socio-economic conditions.

Infants' perinatal variables including gestational age, birth weight, length of stay in the NICU, need for invasive mechanical ventilation (the last two variables were collected only for VPT infants) were obtained from medical records. Neonatal clinical condition was assessed with the Vermont Oxford Network Risk Adjustment index (VON-RA) (Zupancic et al., 2007). The VON-RA index considers clinical and demographical variables, such as gestational age, presence of congenital anomaly, multiple gestation, Apgar score at 1 min, gender, mode of delivery (vaginal vs. cesarean section), and out-born status. Lower VON-RA scores suggest more favorable outcomes in VPT infants.

#### Pain-related stress

Neonatal pain-related stress was quantified according to Grunau and colleagues (Brummelte et al., 2012; Vinall et al., 2012; Grunau et al., 2013). Nonetheless, due to restrictive inclusion criteria adopted in the present work, we selected only VPT infants without severe neonatal morbidities who did not undergo surgery and chest-tube insertions. Consequently, pain-related stress was quantified as the total number of skin-breaking procedures throughout the NICU stay, including heel lance, arterial and venous punctures, peripheral venous line insertion, arterial blood samples.

#### DNA methylation analysis

**Figure 1:**
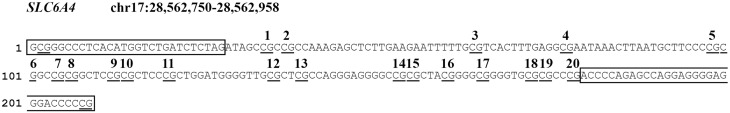
**Schematic overview of the analyzed CpG sites in the *SLC6A4* gene promoter region**. CpG sites are underlined. PCR primer sequences are boxed. Analyzed CpG sites are indicated by numbers 1–20.

We analyzed a CpG-rich region of the *SLC6A4* promoter (chr17:28562750-28562958, Human hg19 Assembly; see Figure 1), between −69 and −213 relative to the transcriptional start site, which contains 20 CpG sites and is adjacent to exon 1a. The region of *SLC6A4* we analyzed has been recognized to be associated with variations in *SLC6A4* mRNA expression (Philibert et al., 2007) and has been previously shown to be differentially methylated in threat-related amygdala reactivity (Nikolova et al., 2014). It contains multiple DNase-hypersensitivity sites (Sabo et al., 2006), a universal feature of active cis-regulatory sequences, and shows the highest degree of evolutionary conservation between 100 vertebrates, measured using phyloP (Siepel et al., 2005) in the 5′-end of the gene. Methylation levels were determined in DNA from cord blood or peripheral blood using bisulfite modification followed by NGS. Genomic DNA was extracted from 0.2 ml of each sample using the GenElute Blood Genomic DNA kit (Sigma). Bisulfite conversion was performed on 500 ng of genomic DNA using the EZ DNA methylation kit (ZymoResearch, Inc, Irvine, CA, USA). Primers were designed using Bisulfite Primer Seeker (http://www.zymoresearch.com/tools/bisulfite-primer-seeker). The gene-specific forward 5′-GYGGGTTTTTATATGGTTTGATTTTTAG-3′ and reverse 5′-CRAAAATCCCTCCCCTCCTAACTCTAAAATC-3′ primers correspond to the boxed sequences in Figure 1. A TruSeq amplicon-specific tail 5′ CCTACACGACGCTCTTCCGATCT 3′ was added to the forward primer, while the sequence 5′ TCAGACGTGTGCTCAACCGATCT 3′ was added to the reverse primer, in order to allow synthesis and sequencing of TruSeq libraries of methylated fragments. Primary PCR amplifications were performed on 20 ng of bisulfite-treated DNA using Taq Gold (Life Technologies, Inc.). Cycling consisted of 5 min pre-activation at 95°C, followed by 35 cycles of 94°C denaturation for 15 s, 58°C annealing for 20 s, 72°C elongation for 1 min 30 s. All PCR products were checked on 2% agarose TAE gels, then treated with Ilustra Exo Pro-STAR (GE Healthcare) to eliminate unincorporated primers. Secondary PCR was conducted on each sample using a TruSeq Custom Amplicon Index Kit (Illumina) containing eight forward (i5) and twelve reverse (i7) index primers allowing unique tagging of 96 samples. Optimal annealing temperature (68°C) and number of PCR cycles (16) were experimentally determined. Cycling consisted of 5 min pre-activation at 95°C, followed by 16 cycles of 94°C denaturation for 15 s, 68°C annealing for 20 s, 72°C elongation for 1 min. Again all PCR products were checked on 2% agarose TAE gels, then approximately equimolar aliquots of each product were pooled and purified on a 2% agarose TAE gel. The purified library was quantified on a Bioanalyzer 2100 (Agilent) and sequenced on a MiSeq (Illumina) using a v2 Reagent kit, 300 cycles PE. Paired ends reads from each sample were independently aligned to all the reference sequences by a parallel striped Smith-Waterman algorithm. Only paired reads that aligned coherently to the same reference sequence were retained. At each CpG site in each sequence, the 4 base frequencies were evaluated and reported along with the C→T percentage.

### Data reduction

#### Pain-related stress

To distinguish levels of pain exposure, VPT infants were divided through median split of the number of skin-breaking procedures (median value = 24): 31 VPT infants were included in the *low pain-exposure* (LPE) group, whereas 25 VPT infants were included in the *high pain-exposure* (HPE) group. This approach had the advantage of avoiding bias from extreme scores.

#### Delta methylation values

In order to assess the variation in methylation percentages from birth to NICU discharge in the VPT group, a *delta methylation value* was obtained for each CpG site subtracting methylation percentage at birth from methylation percentage at NICU discharge. The *delta methylation value* allowed to adjust for possible between-subjects variations in CpG methylation at birth. *Delta methylation values* higher than zero indicated increases in methylation from birth to NICU discharge, whereas *delta methylation values* lower than or equal to zero indicated decreases or no variation.

### Statistical analyses

LPE VPT, HPE VPT, and FT infants were compared for socio-demographic and neonatal variables as well as maternal self-reports through univariate analyses of variance (ANOVAs) and Bonferroni-adjusted *post-hoc*. LPE and HPE VPT infants were compared for the length of stay in the NICU and for the neonatal clinical condition (i.e., VON-RA) through independent-samples *t*-test and for the presence or absence of assisted ventilation through chi-squared test. To evaluate differences at birth in *SLC6A4* DNA methylation between LPE VPT, HPE VPT, and FT infants a multivariate analysis of variance (MANOVA) was used, with Group as between-subject variable (3 levels: LPE VPTs, HPE VPTs, FTs) and percentage of methylation of the 20 CpG sites within the promoter region as dependent variables. To test for *SLC6A4* DNA methylation differences from birth to NICU discharge in the VPT group, a MANOVA was adopted, with Group as between-subject variable (2 levels: LPE VPTs *vs.* HPE VPTs) and the *methylation delta values* for the 20 CpG sites as dependent variables. To control for confounders, any socio-demographic and clinical variable for which significant differences emerged between LPE and HPE VPT infants were included as covariate in a MANCOVA with Group as between-subject variable (2 levels: LPE VPTs *vs.* HPE VPTs) and the *methylation delta values* for the 20 CpG sites as dependent variables. For all analyses, Bonferroni adjusted *post-hoc* tests were used to qualify significant effects as appropriate. Statistical analyses were carried using SPSS 21 for Windows 7 with alpha set at *p* < 0.05.

## Results

### Infant and maternal characteristics

Neonatal variables for infants and socio-demographic variables for mothers are reported in Table 1. Significant differences emerged for gestational age, *F*_(2, 86)_ = 287.55, *p* < 0.001, η^2^_*p*_ = 0.87, and birth weight, *F*_(2, 86)_ = 298.15, *p* < 0.001, η^2^_*p*_ = 0.88. *Post-hoc* tests revealed that full-term infants had higher birth weight than LPE (*p*s < 0.001) and HPE (*p*s < 0.001) VPT infants, whereas LPE and HPE VPT infants did not differ for gestational age and birth weight. Length of stay in the NICU was similar in LPE VPT and HPE VPT infants. No differences in need for invasive mechanical ventilation were observed between LPE and HPE VPT infants. Significant differences emerged between the two groups of VPT infants for the number of skin-breaking procedures, *t*_(54)_ = 3.57, *p* = 0.001 and for VON-RA, *t*_(54)_ = 2.40, *p* = 0.01.

**Table 1:** **Infant and maternal characteristics for low- and high-pain exposed VPT infants and for full-term infants**.

	**Groups**					
	**LPE VPT infants (*N* = 31; *F* = 11)**		**HPE VPT infants (*N* = 25; *F* = 16)**		**FT infants (*N* = 32; *F* = 17)**	
	**Mean**	***SD***	**Mean**	***SD***	**Mean**	***SD***
**INFANTS**						
Gestational age (weeks)	30.71	1.32	30.12	2.24	38.88	1.16
Birth weight (grams)	1445.81	271.44	1356.20	382.08	3352.19	413.43
Length of NICU stay (days)	44.93	17.52	47.88	21.88	—	—
Number of skin-breaking procedures	18.19	4.81	40.80	14.28	—	—
VON-RA score (range:0.01 ÷ 0.99)	0.02	0.01	0.05	0.02	—	—
	***N***	**%**	***N***	**%**	***N***	**%**
Mechanical invasive ventilation	24	77.42	19	76.00	—	—
No mechanical invasive ventilation	7	22.58	6	24.00	—	—
	**Mean**	**SD**	**Mean**	**SD**	**Mean**	**SD**
**MOTHERS**						
Age (years)	35.32	3.75	36.67	5.21	33.57	4.03
Educational level (years of study)	16.81	2.01	15.55	2.44	15.09	3.59
Family SES	65.63	19.31	63.33	20.58	65.78	19.39

LPE VPT, Low-pain exposed very preterm infants; HPE VPT, High-pain exposed very preterm infants; FT, Full-term infants; VON–RA, Vermont Oxford Network–Risk Adjustment index (Zupancic et al., 2007).

### *SLC6A4* methylation at birth

**Figure 2:**
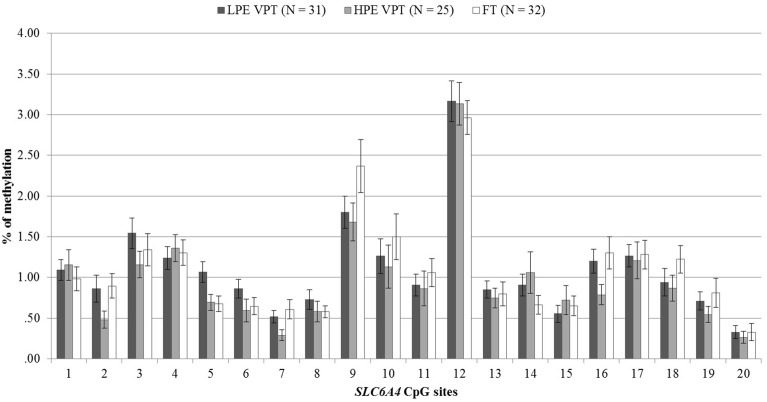
**At-birth methylation percentages of 20 CpG sites within the *SLC6A4* promoter region for low- and high-pain VPT and FT infants**. LPE VPT, Low-pain exposed very preterm infants; HPE VPT, High-pain exposed very preterm infants; FT, Full-term infants.

DNA methylation percentages at birth for the 20 CpG sites included in the promoter region of the *SLC6A4* gene are reported in Figure 2. No differences were observed between the three groups.

### Levels of pain-related stress and *SLC6A4* methylation

**Figure 3:**
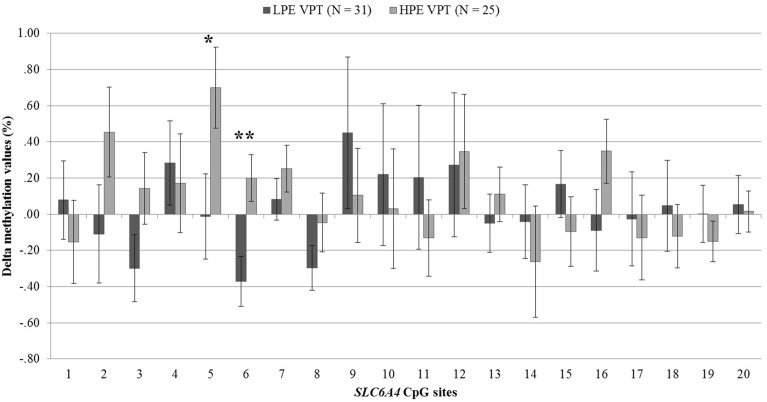
**Delta methylation values from birth to NICU discharge for low- and high-pain exposed very preterm infants**. LPE VPT, Low-pain exposed very preterm infants; HPE VPT, High-pain exposed very preterm infants; ^*^p < 0.05; ^**^p < 0.01.

DNA methylation at birth and NICU discharge for the VPT infants is reported in Table 2. Analyses revealed significant differences in *SLC6A4* methylation delta values between LPE and HPE VPT infants, *F*_(20, 35)_ = 1.91, *p* < 0.05, η^2^_*p*_ = 0.52. Significant differences emerged for CpG site 5, *F*_(1, 54)_ = 4.66, *p* < 0.05, η^2^_*p*_ = 0.08, and site 6, *F*_(1, 54)_ = 8.81, *p* < 0.01, η^2^_*p*_ = 0.14. A significant increase in DNA methylation was found for these sites in the HPE VPT infants, but not in LPE VPT counterpart (Figure 3).

**Table 2:** **Methylation percentages of each of 20 CpG sites within the *SLC6A4* promoter region for low-pain exposed and high-pain exposed very preterm infants at birth and at NICU discharge**.

***SLC6A4* CpG sites**	**Methylation at birth**				**Methylation at NICU discharge**			
	**LPE VPT infants**		**HPE VPT infants**		**LPE VPT infants**		**HPE VPT infants**	
	**Mean**	***SD***	**Mean**	***SD***	**Mean**	***SD***	**Mean**	***SD***
CpG site1	1.09	0.71	1.15	0.95	1.17	0.85	1.00	0.72
CpG site2	0.86	0.92	0.48	0.52	0.79	0.64	0.94	0.51
CpG site3	1.54	1.06	1.16	0.82	1.24	0.92	1.30	0.64
CpG site4	1.24	0.78	1.36	0.83	1.52	0.98	1.53	0.90
CpG site5	1.06	0.71	0.69	0.49	1.05	1.10	1.39	0.98
CpG site6	0.86	0.63	0.59	0.70	0.49	0.42	0.80	0.45
CpG site7	0.52	0.42	0.29	0.33	0.60	0.53	0.54	0.47
CpG site8	0.73	0.67	0.58	0.64	0.43	0.40	0.54	0.44
CpG site9	1.80	1.10	1.68	1.17	2.25	2.09	1.79	1.05
CpG site10	1.26	1.19	1.13	1.33	1.48	2.10	1.16	1.05
CpG site11	0.91	0.75	0.86	1.08	1.11	2.05	0.73	0.38
CpG site12	3.17	1.38	3.14	1.31	3.44	1.61	3.48	1.07
CpG site13	0.85	0.58	0.75	0.61	0.80	0.72	0.86	0.53
CpG site14	0.91	0.74	1.06	1.27	0.86	0.92	0.80	0.69
CpG site15	0.55	0.58	0.72	0.90	0.72	0.67	0.63	0.46
CpG site16	1.20	0.81	0.79	0.62	1.11	0.82	1.13	0.64
CpG site17	1.27	0.76	1.21	1.13	1.24	1.20	1.08	0.74
CpG site18	0.94	0.95	0.87	0.81	0.99	1.03	0.75	0.59
CpG site19	0.71	0.62	0.55	0.50	0.71	0.65	0.40	0.33
CpG site20	0.33	0.44	0.26	0.37	0.38	0.71	0.28	0.41

LPE VPT, Low-pain exposed very preterm infants; HPE VPT, High-pain exposed very preterm infants.

As LPE and HPE VPT infants showed significantly differences in the VON-RA index, a MANCOVA was used to account for potential confounder effect of this variable on the association between levels of pain-related stress and CpG methylation. When adjusting for VON-RA index, the effect of Group remained significant, *F*_(20, 34)_ = 2.04, *p* < 0.05, η^2^_*p*_ = 0.55. HPE VPT infants reported significantly higher delta methylation values for site 5, *F*_(1, 53)_ = 5.14, *p* < 0.05, η^2^_*p*_ = 0.09, and site 6, *F*_(1, 53)_ = 8.92, *p* < 0.01, η^2^_*p*_ = 0.14. No significant effect emerged for VON-RA covariate.

## Discussion

The current study aimed at assessing DNA methylation at 20 CpG sites within the promoter region of the *SLC6A4* gene in relation to different levels of pain-related stress during NICU stay in VPT infants. Also, we controlled for methylation levels at birth comparing FT and VPT infants.

No differences emerged in *SLC6A4* methylation at birth between VPT and FT infants, suggesting a lack of significant relationship between birth status (i.e., preterm vs. full-term birth) and epigenetic markers in the stress-related gene encoding for serotonin transporter. These findings are consistent with previous research on the epigenetic correlates of preterm birth, which documented no differences in the methylation status of imprinted genes between preterm and full-term infants at birth (Liu et al., 2013). The present study suggests that preterm birth does not associate *per sé* with alterations of a specific stress-related gene, namely the *SLC6A4*, which has documented associations with stress regulation later in life (Lesch, 2011). Nonetheless, it should be noted that a recent study found differences in the methylation rates of the gene encoding for glucocorticoid receptors (i.e., *NR3C1*) between preterm infants and full-term infants at birth (Kantake et al., 2014). Thus, it is quite plausible that birth status might differently affect DNA methylation of diverse stress-related genes. Consistently, further research is warranted to establish to which extent preterm birth *per sé* might exert epigenetic alterations on specific stress-related genes.

In the VPT group, an increase of methylation percentage was observed from birth to discharge at two specific CpG sites (i.e., site 5 and site 6) only for infants exposed to high levels of pain-related stress during NICU stay. This result is consistent with a recent retrospective study which has documented that the exposure to pain-related stress during NICU stay was associated with changes in specific CpG sites of the *SLC6A4* gene in 7-year-old VPT children (Chau et al., 2014). Importantly, children enrolled in the just mentioned study were not assessed for *SLC6A4* methylation status at NICU discharge. Although Chau et al. (2014) controlled for neonatal clinical confounders, it cannot be ruled out that the epigenetic changes observed at age 7 might be affected by factors other than pain exposure during NICU stay, such as life events during infancy and childhood. What is unique of our study is that LPE and HPE VPT infants were not different for socio-demographic characteristics (e.g., socio-economic status), clinical variables (e.g., gestational age and birth weight), and environmental factors (e.g., length of stay in the NICU), ruling out the plausible impact of additional stressful events other than pain-related stress experienced by VPT infants during the hospitalization. Although, HPE VPT infants had higher a VON-RA clinical status score, when analyses have been adjusted for the VON-RA the significant effect of high levels of pain-related stress on CpG sites 5 and 6 remained significant and no effect of VON-RA on DNA methylation of *SLC6A4* emerged. Overall, findings from the present study suggest an altered *SLC6A4* methylation status at NICU discharge which is attributable, at least partially, to the amount of pain-related stress experienced by VPT infants during NICU stay.

The current results might help in understanding the epigenetic pathways through which early adversity in the form of high levels of pain-related stress experienced during the NICU stay might be embedded in the developing biology of VPT infants. Importantly, it is well-known that high levels of pain-related stress exposure in VPT infants are associated with a cohort of alterations in cerebral, physiologic, and behavioral development during childhood and adulthood, heightening the risk of behavioral and affective difficulties later in life (Brummelte et al., 2012; Grunau et al., 2013; Ranger et al., 2013). Interestingly, alterations in the epigenetics of the *SLC6A4* gene have recently, been linked with similar developmental outcomes. Altered *SLC6A4* methylation has been associated with reduced hippocampal gray matter (Dannlowski et al., 2014) and HPA cortisol reactivity in human adults (Alexander et al., 2014). Furthermore, a recent report has documented the association between depressive symptoms and heightened *SLC6A4* methylation at 10 of the same 20 CpG sites assessed in the present work (Zhao et al., 2013). In the light of this evidence, the transcriptional activity of the *SLC6A4* appears to be specifically susceptible to epigenetic alterations in response to early adverse experiences, leading to a number of functional and structural alterations throughout infants and children development. From this point of view, it seems plausible to speculate that the altered methylation status observed in this study for high-pain exposure infants might potentially contribute to put these infants at a major risk condition for adverse outcomes during infancy and childhood. Further, research is warranted to investigate specific associations between NICU-related methylation changes and subsequent neurobehavioral development in preterm infant.

This study has some limitations. First, the analyses were performed on different samples at birth (i.e., cord blood for both VPT and FT infants) and at NICU discharge for preterm infants (i.e., peripheral blood). Cord blood sampling has been already used in newborn epigenetic studies (Oberlander et al., 2008) and it has been suggested that mean methylation values of umbilical cord blood cells resembled those from peripheral blood cells in healthy individuals (Tabano et al., 2010). Second, previous researches documented that structural (i.e., polymorphism) and functional (i.e., DNA methylation) modifications of *SLC6A4* might jointly affect the behavioral developmental outcome after stress exposure (Van Ijzendoorn et al., 2010, 2012; Montirosso et al., 2015b). Thus, caution is needed when interpreting the present findings, as it is important to consider that multiple genetic mechanisms may contribute to behavioral development and temperament. Although an exploration of the interaction between *5-HTTLPR* and *SLC6A4* methylation was not an objective of the present investigation, we acknowledge that future studies should address this joint influence in the context of preterm infants' development. Third, this study was focused on preterm infants without major clinical complications. As such, several pain-related stressors connected with NICU stay (e.g., chest-tube insertion, abdominal punctures, and prolonged need for invasive mechanical ventilation) did not apply to subjects enrolled in the present work. Future studies are warranted to account for differential epigenetic effects of diverse stressors among populations of preterm infants at different clinical and developmental risk. Finally it should be noted that bisulfite conversion analysis does not differentiate between 5 mC and 5 hmC residues. As 5 mC and 5 hmC have different implications for early biological development and gene transcription regulation, future studies are suggested to adopt tools capable to unequivocally distinguish 5 mC and 5 hmC, such as digestion of *in vitro* synthesized double-stranded oligonucleotides (Gao et al., 2013).

With the ongoing reduction of mortality in preterm neonates, the focus of neonatal health care systems is shifting from granting survival to sustaining functional outcomes through improved developmental care (Als et al., 2004, 2012; Montirosso et al., 2012). Consistently, the study of epigenetic variations associated with prematurity might not only provide new insights in the identification of biological markers associated with long-term changes in stress reactivity, but it can be used as a measure of the quality of developmental care and early intervention programs in neonatal period (Samra et al., 2012). The findings reported in the current study add to the emerging literature in Preterm Behavioral Epigenetics (Provenzi and Montirosso, 2015), highlighting the importance of assessing stress-related epigenetic markers in very preterm infants exposed to high levels of pain.

### Conflict of interest statement

The authors declare that the research was conducted in the absence of any commercial or financial relationships that could be construed as a potential conflict of interest.
